# Investigation of genetic diversity and population structure of common wheat cultivars in northern China using DArT markers

**DOI:** 10.1186/1471-2156-12-42

**Published:** 2011-05-11

**Authors:** LiYi Zhang, DongCheng Liu, XiaoLi Guo, WenLong Yang, JiaZhu Sun, DaoWen Wang, Pierre Sourdille, AiMin Zhang

**Affiliations:** 1 The State Key Laboratory of Plant Cell and Chromosome Engineering, Institute of Genetics and Developmental Biology, Chinese Academy of Sciences, Beijing, China; 2 Guizhou Institute of Dryland Crops, Guizhou Academy of Agricultural Sciences, Guiyang, China; 3 College of Biological Sciences, China Agricultural University, Beijing, China; 4 UMR INRA-UBP Amélioration et Santé des Plantes, Clermont-Ferrand Cedex 2, France

## Abstract

**Background:**

In order to help establish heterotic groups of Chinese northern wheat cultivars (lines), Diversity arrays technology (DArT) markers were used to investigate the genetic diversity and population structure of Chinese common wheat (*Triticum aestivum *L.).

**Results:**

In total, 1637 of 7000 DArT markers were polymorphic and scored with high confidence among a collection of 111 lines composed mostly of cultivars and breeding lines from northern China. The polymorphism information content (PIC) of DArT markers ranged from 0.03 to 0.50, with an average of 0.40, with P > 80 (reliable markers). With principal-coordinates analysis (PCoA) of DArT data either from the whole genome or from the B-genome alone, all lines fell into one of two major groups reflecting 1RS/1BL type (1RS/1BL and non-1RS/1BL). Evidence of geographic clustering of genotypes was also observed using DArT markers from the A genome. Cluster analysis based on the unweighted pair-group method with algorithmic mean suggested the existence of two subgroups within the non-1RS/1BL group and four subgroups within the 1RS/1BL group. Furthermore, analysis of molecular variance (AMOVA) revealed highly significant (*P *< 0.001) genetic variance within and among subgroups and among groups.

**Conclusion:**

These results provide valuable information for selecting crossing parents and establishing heterotic groups in the Chinese wheat-breeding program.

## Background

Bread wheat (*Triticum aestivum *L.), an economically important cereal, is widely cultivated worldwide. Of the nearly 600 Mt of wheat harvested worldwide, about 80% is used as human food [1]. Wheat-breeding programs around the world are working toward improved grain yield with better quality, disease-resistance and agronomic performance.

Knowledge of the genetic diversity within a germplasm collection is the basis for selection of crossing parents, establishing heterotic groups and has a significant impact on the improvement of crops. Therefore, assessment of the extent and nature of genetic variation in bread wheat is important to breeding and genetic resource conservation programs. As molecular markers have been developed, they have been extensively explored for analysis of genetic diversity in common wheat; such markers include restriction fragment length polymorphisms (RFLPs) [2,3], randomly amplified polymorphic DNA (RAPD) [4,5], amplified fragment length polymorphisms (AFLP) [6,7], and simple-sequence repeats (SSR) [8-12].

The 1RS/1BL translocation is one of the most frequently used alien introgressions in wheat-breeding programs throughout the world [13,14]. In addition to its advantage in disease resistance (Sr31, Lr26, and Yr9) [15], the 1RS translocation is also useful for its positive effect on agronomic traits including yield performance, yield stability, and wide adaptation [16,17]. However, 1RS carries the Sec-1 locus coding for ε-secalin, which results in negative effects on bread-making quality, such as poor mixing tolerance, superficial dough stickiness, and low bread volume [18,19]. Some defects in noodle processing quality are also correlated with this translocation [20,21]. Various molecular markers, including STS (Sequence-Tagged Site), SCAR (Sequence Characterized Amplified Region), RAPD, and SSR, have been employed to detect the 1RS/1BL translocation [22-24]

Recently, diversity arrays technology (DArT) markers were developed to discover and score genetic polymorphic markers in the whole genome. This technology is a sequence-independent and high-throughput method [25]. DArT markers have been applied to several species including cereals such as barley (*Hordeum vulgare *L.), wheat (*Triticum aestivum *L.), and durum wheat (*Triticum durum *L.). Not only the technology has been used to create high-density genetic maps [26] and for association studies [27,28], but it is also expanding into the study of genetic diversity and population genetics [29-31]. Stodart et al. [30] compared AFLP, SSR, and DArT markers and found that DArT markers are suitable for estimation of genetic diversity in landrace cultivars of bread wheat.

China is the world's most populous nation, accounting for 20% of the global population. Crop yield has always been a primary concern of agronomists in China. Northern wheat production plays an important role in China's food production. Production in the major growing area of northern China, represents 45-53% of wheat acreage, and accounts for 47-61% of the total production [32]. Since 1RS translocations were introduced to China in the early 1970s, they have been widely used in wheat-breeding programs [33]. In some major wheat-growing area of northern China, 1RS/1BL translocations are present in 50 ~ 70% of all wheat cultivars [34]. Therefore, information about the genetic diversity and the distribution of 1RS/1BL translocations within Chinese germplasm is very important for improving wheat yield with better quality in Chinese northern breeding programs.

To help establish heterotic groups of Chinese northern wheat cultivars (lines), we assessed the genetic diversity in a collection of cultivars from northern China using DArT markers and diversity analysis. We also used DArT data to investigate the distribution of 1RS/1BL lines within the northern cultivars.

## Results

### Polymorphism of DArT markers

Scanning with about 7000 DArT markers resulted in 2264 polymorphic loci, of which 1362 markers were assigned to the 21 wheat chromosomes. Each DArT marker was subjected to an ANOVA-based statistical analysis to estimate its quality, described as a P-value (the between-allelic-states variance of the relative target hybridization intensity as a percentage of the total variance). In general, markers with *P*-values above 80 were scored very reliably, according to Triticarte Pty. Ltd. Finally, 1637 DArTs were selected with P-values > 80, 1009 markers of them had genetic locations on the wheat chromosomes. Most of the 1009 DArTs were located on the B genome (566), followed by the A (290) and D genomes (88). Similarly, these DArTs were unevenly distributed among the seven homologous groups of common wheat chromosomes. Group 1 harbored the largest number (241), followed by group 3 (182), group 6 (141), group 7 (138), group 2 (133), group 5 (91), and group 4 (83).

Polymorphism information content (PIC) values, computed for the 1637 polymorphic markers, ranged from 0.03 to 0.50 (the maximum value for a biallelic marker), with a mean value of 0.40. DArT allele frequencies ranged from 0.22 to 0.78 (mean = 0.43 ± 0.13) and showed a near-normal distribution. The DArT markers used showed different levels of genetic diversity; Nei's genetic diversity [35] ranged from 0.11 to 0.51, with an average of 0.40 for all markers. As this index is equivalent to the PIC index, these results also provide an estimate of the discriminatory power of each DArT locus.

### Population structure on the whole-genome level

**Figure 1:**
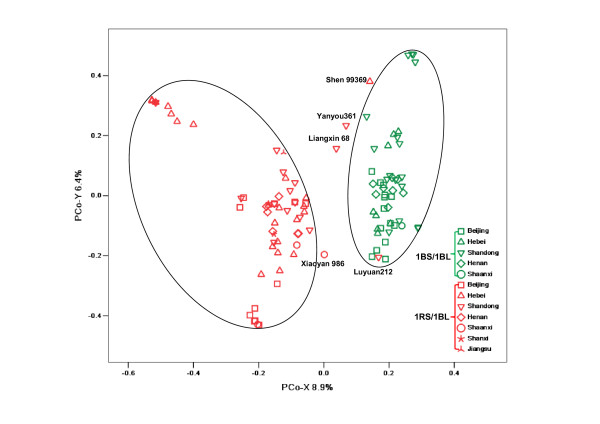
**Distribution of varieties in two-dimensional PCoA space based on DArT data at the whole-genome level**. Non-1RS/1BL lines are in green type, and 1RS/1BL lines are in red.

PCoA analysis was used to examine population structure in the collection, using the 1637 DArT markers (P > 80%) distributed throughout the entire genome. A two-dimensional scatter plot of the 111 wheat genotypes, shown in Figure 1, reveals two clear groups. The first (PCo-X) and second (PCo-Y) principal coordinates accounted for 8.9% and 6.4% of the variation, respectively. The right-hand group proved to be highly clustered around the PCo-X axis with no obvious separation of cultivars (lines) originating from different geographic regions. In contrast, the left-hand group was spread widely along both the PCo-X and PCo-Y axes. Most genotypes from Hebei province was located in the upper left quadrant, some genotypes from Shandong clustered in the upper right quadrant, and some from Beijing falling into the lower left quadrant.

### Between-population differences

Linkage disequilibrium analysis was performed to investigate differences between the two groups. As a result, 136 DArT markers on chromosome 1B showed significant LD between DArT markers and the "group locus" with an average *r*^2 ^value of 0.69 (*P *< 0.001; range: 0.1 to 1). Among these markers, 11 showing complete LD (*r*^2 ^= 1 with *P *= 0) were observed on chromosome 1BS. Considering the possible existence of 1RS/1BL translocations in Chinese cultivars (lines), the primers specific for 1RS of rye were subsequently used to scan all cultivars (lines); this demonstrated that our collection was distinctly separated based on 1RS/1BL and non-1RS/1BL translocations with a few exceptions (see Additional file 1). As shown in Figure 1, the right-hand group was composed of 47 non-1RS/1BL lines, and the left-hand group of 61 1RS/1BL lines. Four 1RS/1BL lines, i.e., cv. Xiaoyan986, Liangxin68, Yanyou361, and Shen99369, were scattered between the two groups. Only one 1RS/1BL line (Luyuan212) fell within the 1BS/1BL cluster.

### Population structure based on different wheat genomes

Differences in the genetic structure among the three genomes, A (290), B (566), and D (88), were investigated for relationships among these cultivars (lines).

**Figure 2:**
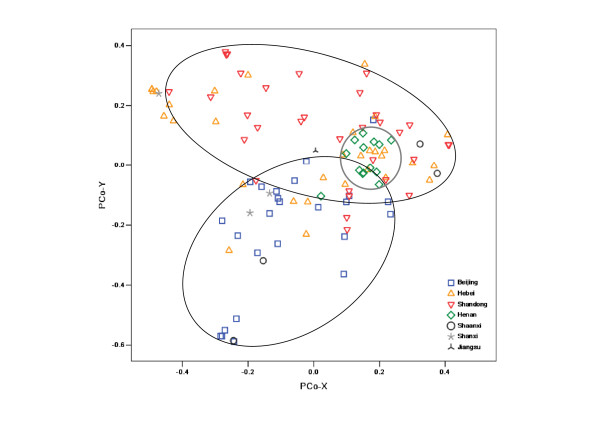
**Distribution of varieties in two-dimensional PCoA space (A genome)**.

PCoA analysis based on genotype data from the 290 DArT markers on genome A showed that our collection was roughly divided into three groups according to geographical origin (Figure 2). The majority of cultivars (lines) from Beijing grouped together, whereas another group mainly contained cultivars from Shandong province. Cultivars (lines) from Henan province were clustered together at the junction of the Beijing and Shandong groups. However, cultivars (lines) originating from Hebei province did not separate out, and were scattered among the three groups.

**Figure 3:**
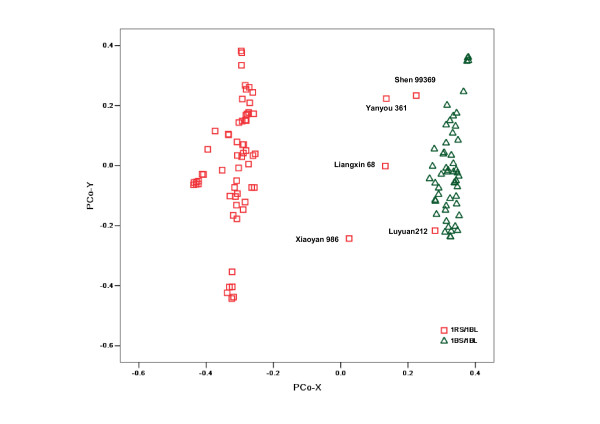
**Distribution of varieties in two-dimensional PCoA space (B genome)**.

Similarly, PCoA analysis was performed based on genotype data from the 566 DArTs on genome B. A clear separation of 1RS/1BL and non-1RS/1BL lines was observed with the sole exception of Luyuan212, which was consistent with the result obtained using DArTs from the whole genome. Similarly, the four 1RS/1BL lines were distributed between the two groups and close to the 1BS/1BL groups (Figure 3).

**Figure 4:**
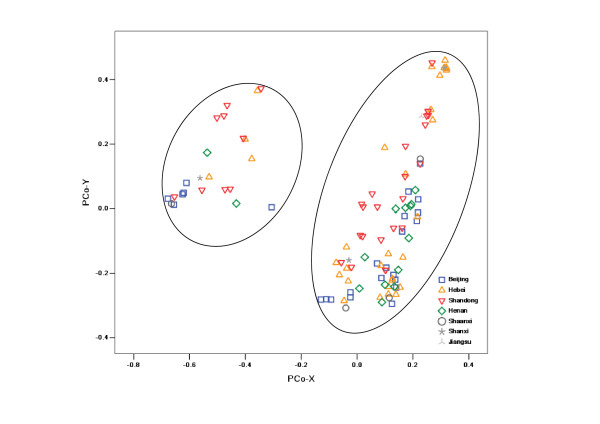
**Distribution of varieties in two-dimensional PCoA space (D genome)**.

PCoA analysis based on the 88 DArT markers on the D genome showed a different profile. The separation was not based on geographical origin or 1RS/1BL translocations. All cultivars (lines) fell into two distinct groups: a large group comprising 88 lines and a small group of the remaining 23 lines. The components of the small group mainly originated from Shandong and Beijing (Figure 4). In the same way, LD analysis performed to investigate the relationship between the two groups, showed that 20 DArTs on chromosome 1D presented significant LD between the marker and the "group locus" with an average *r*^2 ^value of 0.95 (*P *< 0.001)

### Genetic relationships among wheat cultivars

**Figure5:**
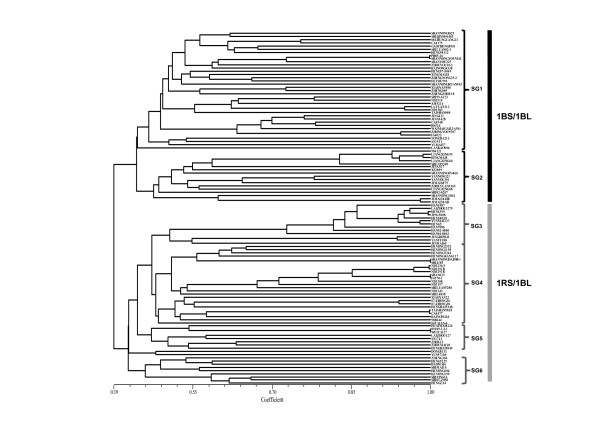
**Dendrogram representing the relationships among the 111 Chinese wheat cultivars**. That was revealed by UPGMA cluster analysis based on Jaccard genetic distances. Non-1RS/1BL lines are in black type, and 1RS/1BL lines are in grey.

The Jaccard genetic distance coefficients (d) ranged from 0 to 0.76, with an overall mean of 0.55. The greatest distance was observed between the cultivars cv. Shi01-Z056 and Jinmai60. The d-values did not differ greatly between cultivars (lines) with different origins (0.51 for cultivars from Beijing and 0.56 for those from Hebei). A consensus dendrogram obtained from UPGMA of d was constructed and is shown in Figure 5. Again, a clear separation of 1BL/1RS and non-1BL/1RS types was observed. Two clear sizable (>5 genotypes) subgroups (SG1 and SG2) within the group formed by non-1RS/1BL lines can be distinguished. SG1 was composed of lines from all regions, whereas the lines within SG2 were primarily from Shandong province. Within the 1RS/1BL branch of the tree, genotypes fell into four subgroups (SG3-SG6). SG3 consists of 27 cultivars that were predominantly from the Hebei province, whereas SG4 primarily contained 30 cultivars that were exclusively from the Beijing region. The final two subgroups (SG5 and SG6) mainly consisted of cultivars from Heibei province.

### Analysis of molecular variance (AMOVA)

As described above, six important subgroups were identified in the cluster analysis, and these were used to perform an AMOVA on the collection of lines. The collection was considered to be composed of two groups (based on 1RS-type) and six subgroups. Highly significant (*P *< 0.001) genetic variance was observed within and among subgroups and among groups (Table 1). The variance within subgroups accounted for the largest portion (72.2%) of total variance, whereas 9.3% of the variance was observed among groups, and differences among subgroups contributed 18.5% of the total variance. The estimated fixation index (F_ST_) value of 0.28 suggests that this germplasm was highly differentiated. Subgroup pairwise F_ST _results showed that the lowest (0.11) and the highest (0.42) F_ST _values were present between subgroups 4 and 5 and between subgroups 2 and 3, respectively (Table 2).

**Table 1:** Analysis of molecular variance for 111 wheat cultivars in northern China

Source of variation	d.f.	Sum of squares	Variance components	V%
Among 1RS-type groups	1	3191.44	29.35	9.36
Among subgroups within 1RS-type groups	4	4515.87	57.98	18.49
Within subgroups	103	23310.76	226.32	72.15
Total	108	32018.07	313.66	
Fixation index (F_ST_) = 0.28				

**Table 2:** Population pairwise F_ST _values

Subgroup	1	2	3	4	5
1					
2	0.18				
3	0.36	0.42			
4	0.20	0.33	0.30		
5	0.20	0.23	0.29	0.11	
6	0.25	0.32	0.40	0.19	0.13

## Discussion

### DArT markers

In this study, we demonstrated the power of DArT markers for investigating population structure and genetic diversity in common wheat; these markers will be useful in integrating this work with future objectives and marker-based results in the wheat-breeding program. DArT markers proved cost effective and ideally suited to high-throughput parallel analysis that was not dependent on in-house infrastructure. These results will be cross applicable with many other projects in wheat that are also using DArT-based markers. Furthermore, when DArT clone sequences are available, these will provide access to other methods for marker design and for orthology-based comparison among species. A recent analysis of DArT clone sequences in oat showed that over one-third of these markers contain DNA sequences with strong homology to functionally annotated genes [36].

DArT markers in the present study showed an average PIC of 0.40, which suggests that they are sufficiently informative. The polymorphic DArT markers in our samples were frequently present on the B genome, but seldom occurred on the D genome, in keeping with the relative lack of polymorphic DArT markers found in this genome by Akbari et al. (2006). This agrees with the fact that the B genome presents the highest level of polymorphism, whereas the D genome shows the lowest level among the wheat ABD genomes [37-39].

### IRS/1BL translocation in Chinese cultivars

STS, SCAR, RAPD, and SSR molecular markers have been employed to detect the 1RS/1BL translocation [24]. DArT markers are dominant, and their polymorphism is based on SNPs and INDELs at restriction enzyme cleavage sites and restriction fragments [31]. DArT markers were shown in this study to be a very powerful tool for detecting 1RS/1BL translocation. PCoA results, based on data from 1637 DArTs from the entire genome, agreed with those obtained by 1RS-specific markers [40] with an exception of Luyuan212. When the DArTs on chromosome 1B that contributed to separating the translocation lines were removed, the separation was no longer observed, and the profile was similar to that corresponding to the A genome (Figure not shown). In comparison with other molecular markers, DArT was a useful method not only for distinguishing 1RS/1BL lines, but also for possibly identifying the origins or the size of translocation fragments. In this study, several 1RS/1BL lines were observed scattered between the 1RS/1BL and non-1BS/1BL groups, two of which were derived from Shandong province. This result suggests that these lines are likely to carry 1RS with different sizes of 1RS segments or different origins of the 1RS chromosome [24]. Further work including cytological observations is needed to confirm this possibility.

Additional information was revealed by DArT markers based on the different genomes used. Our collection was approximately grouped by geographical region when using data from DArTs on the A genome. Cultivars formed three groups: Beijing, Henan, and Shandong. This clustering result may be explained by the presence of some important development genes on the A genome, which may be involved in selection for adaptation to local environments. For example, chromosome 5A is known to carry a number of genes affecting adaptability and productivity, such as the vernalization requirement gene Vrn-A1, one of the main determinants of the winter/spring growth-habit polymorphism [41]. In addition, the ear morphology gene q, the frost-resistant gene Fr1 [42], and a QTL response to environmental stress [43] have all been located on the long arm of 5A. Therefore, cultivars bred in similar ecological zones may have similar adaptability and therefore group together. However, the cultivars from Hebei province overlapped with those from other regions; this may be explained by the fact that Hebei province is located at the junction of these three areas, and therefore, the Hebei cultivars (lines) may show extensive genetic diversities. In our collection, Hebei's genotypes were more diverse than those from the other regions at the A-genome level; the decreasing order of average genetic diversity was Hebei (0.59) > Shandong (0.57) > Henan (0.56) > Beijing (0.53). PCoA analysis performed using data produced by a set of DArT markers assigned to the B genome of common wheat produced a similar result to that based on the whole genome. This is fully explained by the fact that our collection was clustered based on markers specific to translocation of 1RS/1BL on chromosome 1B. In addition, significantly different clusters were observed in the PCoA profile of DArT markers on the D genome; 23 cultivars (lines) that split from the others were mainly composed of genotypes from Shandong and Beijing. Through LD analysis, we found that this separation depended on the 20 DArT markers on chromosome 1D. However, the distribution of DArT markers on the D genome is largely skewed toward chromosome 1D (nearly half, 39/88). These deviated markers are likely to lead to such a clustering, but future cytological analysis will be required to determine the reason for the 1RS/1DL translocation.

As reported by Zhang et al [34], 1RS/1BL translocation accounts for up to 50-70% of all wheat cultivars in some major wheat-growing area of China. In our study, the results from UPGMA cluster and PCoA analysis revealed that 63.6% (64/111) of wheat cultivars (lines) were 1RS/1BL lines. The distribution of 1RS/1BL translocation was different in the Northern China. The increasing order of percentage of 1RS/1BL lines was Beijing (46%, 11/24) < Shandong (47%, 15/32) < Henan (50%, 7/14) < Hebei (73%, 24/33) in our collection. Only 25% of genotypes from Dezhou in Shandong province were 1RS/1BL translocation lines, while all cultivars (lines) from Baoding (6) and Handan (4) in Hebei province were 1RS/1BL lines (Additional file 1). As mentioned above, Hebei's genotypes showed more genetic diversity than those from the other regions. The introduction of 1RS/1BL increased the genetic diversity but brought in some inferior traits, because the ε-secalin coded by Sec-1 locus on 1RS results in negative effects on bread-making and noodle processing quality. With the improvement of living standards in China, the demand for high-quality wheat has been growing; thus, improving wheat quality has recently become an important goal of the Chinese wheat-breeding program. Information about the genetic diversity and the distribution of 1RS/1BL translocations within a germplasm collection is very important for establishing heterotic groups and improving breeding efficiency. To obtain high-quality cultivars (lines), Chinese breeders should avoid selecting crossing parents from the same 1RS/1BL group.

## Conclusions

Our studies demonstrated that DArT markers are a powerful tool for investigating population structure and genetic diversity in common wheat. We separated 111 Chinese wheat cultivars (lines) into two distinct groups based on whether they carry 1RS or non-1RS segments using DArT markers from both the whole genome and the B genome. Our analysis resulted in three geographical groups of cultivars at the A genome level: Beijing, Shandong, and Henan. This information is very important for wheat breeding in China.

## Materials and methods

### Plant materials

A set of 111 common wheat cultivars and breeding lines was selected to represent the germplasm use in northern China during the last decade. These cultivars (lines) were high quality or high yield, including 24 from the Northern winter wheat region (Beijing region), 87 from the Yellow-Huai winter wheat region (Hebei, Henan, Shandong, Shaanxi, Shanxi, and Jiangsu provinces). A complete listing of these genotypes is provided in Additional file 1.

### DArT genotyping and 1RS/1BL detection

DNA was extracted from young leaf tissue from a single plant of each genotype using the protocol recommended by Triticarte Pty. Ltd. (http://www.triticarte.com.au). A total of 111 DNA samples were sent to Triticarte for DArT analysis using the common wheat PstI(TaqI) v3.0 array, which comprises 19,000 clones known to be polymorphic for about 7000 markers in a wide range of wheat cultivars, and about 3500 markers have been assigned to chromosomes using nulli-tetrasomic (NT) lines derived from Chinese Spring [39].

1RS/1BL lines were detected with five 1RS-specific insertion-site-based polymorphism (ISBP) markers [40]. The five primer pairs are listed in Table 3. PCR was performed according to the protocol described in Bartos et al.[40].

**Table 3:** List of 1RS-specific ISBP markers

Marker	Left primer	Right primer	Annealing temp. (°C)
ora013	TGTTATATGAGCGCGAACCA	GCAGAAGTTGGGCGTGTACT	62
ora014	AACTCCGAATCGTTGGGATA	CCGTCGTCCCAAAATAGTGT	62
ora015	GCCGTCGTCTTCCTGAATAG	ACCAAGATGAGCACCAAACC	62
ora016	TCCGTCCCCGTCTCCGTC	ATAAGCCGTGCAAGTCGCC	62
ora017	TGACAAATTGGTTCGCAAGGGG	GCAGCCGTCCACAGACATATAG	62

### Statistical analyses

The polymorphism information content (PIC) values were calculated for each DArT marker using the formula PIC = 1 - ∑ (P*i*)^2^, where P*i *is the proportion of the population carrying the *i*th allele [44]. Nei's genetic diversity is defined as the probability that two randomly chosen haplotypes are different in the sample and was estimated with the formula [35,45].

A binary matrix was produced from the DArT data by scoring fragments as 1 or 0 for the presence or absence of a specific marker allele, respectively. Because DArT markers were scored as dominant, no attempt was made to identify loci harboring heterogeneous or heterozygous alleles. Consistent 0/1 data matrices were used as input for genetic diversity and population structure analysis. NTSYSpc (version 2.0) analysis software was used to perform principal-coordinates analysis (PCoA) using a genetic similarity matrix based on the Jacard genetic similarity index (s_ij_)[46]. The Jacard coefficient (s_*ij*_) measures the asymmetric information on binary variables and is computed according to the following formula: s_*ij *_= *p*/(*p+q+r*), where *p *= number of bands present in both individuals (*i *and *j*), *q *= number of bands present in *i *and absent in *j*, *r *= number of bands present *j *and absent in *i*. Based on decomposition of any multidimensional distance metric, PCoA analysis is similar to the more familiar principal-components analysis (PCA), which is based on Euclidean coordinates. The NTSYSpc analysis software was also used to construct an unweighted pair-group method with algorithmic mean (UPGMA) dendrogram.

Linkage disequilibrium analysis was performed to investigate differences between the two groups. Each classified group was defined as a "locus," with the cultivars (lines) in one group scored as "0" and those in another group scored as "1." Linkage disequilibrium (LD) between pairs of polymorphic loci was evaluated using the software package TASSEL1.9.4 (http://www.maizegenetics.net/). LD was estimated using the squared allele frequency correlation (*r*^2^), which is a measurement of the correlation between a pair of variables [47].

In addition, analysis of molecular variance (AMOVA) was used to estimate the genetic structure among groups and subgroups of cultivars. This method works on a distance matrix between samples in order to measure the genetic structure of the population from which the samples are drawn. It was carried out using ARLEQUIN v3.11 [48] to estimate genetic variance components and to partition the total variance within and among subgroups and among groups. The significance of variance components was tested using 1000 permutations. The fixation index (FST), which is a measure of population differentiation and genetic distance, based on genetic polymorphism data, was computed.

## Authors' contributions

LZ carried out the molecular genetic studies, performed the statistical analysis and drafted the manuscript. DL and XG participated in the design of the study. JS carried out the field test. WY participated in the molecular genetic studies. DW, SP and AZ contributed to revisions for the final draft. AZ conceived of the study, and participated in its design and coordination. All authors read and approved the final manuscript.

## Supplementary Material

Additional file 1 **Common wheat cultivars and lines in northern China used in this study**. Additional file provides information of cultivars (lines), including its origin and pedigrees, cultivated in Chinese wheat region, and carrying or not 1RS/1BL translocation. In the table, symbols '**+**' and '-' indicate the presence and absence of 1RS/1BL, respectively. Click here for file
